# THE ACHIEVE TRIAL: EFFECT OF HEARING LOSS INTERVENTION ON WHITE MATTER INTEGRITY

**DOI:** 10.1093/geroni/igae098.0054

**Published:** 2024-12-31

**Authors:** Jennifer Deal

**Affiliations:** Johns Hopkins University, Baltimore, Maryland, United States

## Abstract

Hearing treatment delays cognitive decline in at-risk older adults. Pathways linking hearing loss to cognitive decline are unknown but may include central effects on brain structure/function. The ACHIEVE randomized controlled trial tested hearing intervention (amplification and rehabilitation) versus health education control on cognitive decline in 977 adults aged 70-84 years. Here we describe the effect of treatment on the pre-specified outcomes of white matter hyperintensity (WMH) volume and microstructural integrity in 445 participants who underwent 3T brain magnetic resonance imaging scans at baseline and Year 3. Fractional anisotropy (FA) and mean diffusivity (MD) were derived from diffusion tensor imaging. Participants were 76.4±4.0 years, 50% female and 12% self-reported Black race. Control (vs. hearing intervention) participants were older and had lower baseline memory scores, but otherwise treatment groups were balanced on measured characteristics. Among 303 participants who completed both MRI scans (n=141 treatment, n=162 control), using linear mixed effects models in an intention-to-treat analysis adjusted for baseline hearing loss severity, recruitment source, study site, age, sex, education, interaction terms between each covariate and time, and time-varying total intracranial volume, hearing treatment slowed rates of WMH volumetric change in whole brain (difference in 3-year change comparing intervention to control: -0.077 cm3, 95% confidence interval [CI]: -0.152, -0.002), with the strongest lobar effect in the occipital lobe (-0.151, 95% CI: -0.284, -0.017). No consistent associations were observed for FA and MD. Follow-up of ACHIEVE participants is ongoing to determine if intervention effects on white matter integrity require longer follow-up time.

